# Impact of COVID-19 on healthcare-associated infections: Antimicrobial consumption does not follow antimicrobial resistance

**DOI:** 10.1016/j.clinsp.2023.100231

**Published:** 2023-06-13

**Authors:** Maristela Pinheiro Freire, Denise Brandão de Assis, Bruno de Melo Tavares, Valquiria O.C. Brito, Igor Marinho, Milton Lapchik, Ana Rubia Guedes, Geraldine Madalosso, Maura Salaroli Oliveira, Antonio Carlos Pedroso de Lima, Anna S. Levin

**Affiliations:** a Department of Infection Control of Hospital das Clínicas, Faculdade de Medicina, Universidade de São Paulo, São Paulo, SP, Brazil; b Division of Hospital Infections, Center for Epidemiologic Surveillance “Prof. Alexandre Vranjac”, Center for Disease Control, Sao Paulo State Health Department, São Paulo, SP, Brazil; c Núcleo Municipal de Controle de Infecção Hospitalar, São Paulo City Health Department, São Paulo, SP, Brazil; d Department of Infectious Diseases, Laboratório de Investigacao Médica ‒ LIM 49, and Instituto de Medicina Tropical, Universidade de São Paulo, São Paulo, SP, Brazil; e Department of Statistics, Institute of Mathematics and Statistics, Universidade de São Paulo, São Paulo, SP, Brazil

**Keywords:** Multidrug-resistance, COVID-19, Antimicrobial consumption, Pneumonia, Bloodstream infection

## Abstract

**Background:**

This study aimed to analyze the Healthcare-Associated Infections (HAI) rates and antimicrobial consumption in Intensive Care Units (ICU) in São Paulo city during the COVID-19 pandemic and compare them with the pre-pandemic period.

**Methods:**

This cohort included all hospitals that reported HAI rates (Central-Line-Associated Bloodstream Infection ‒ CLABSI and Ventilator-Associated Pneumonia ‒ VAP), the proportion of microorganisms that caused CLABSI, the proportion of resistant microorganisms, and antimicrobial consumption from January 2017 ‒ December 2020. Hospitals were stratified by the number of beds, Central Venous Catheter (CVC) utilization rate, Mechanical-Ventilation (MV) utilization rate, and type of funding. Statistical analyses were based on time-series plots and regression models.

**Results:**

220 ICUs were included. The authors observed an abrupt increase in CLABSI rates after the pandemic onset. High CLABSI rates during the pandemic were associated with hospital size, funding (public and non-profit private), and low CVC use (≤ 50%). An increase in VAP rates was associated with public hospitals, and high MV use (> 35%). The susceptibility profile of microorganisms did not differ from that of the pre-pandemic period. polymyxin, glycopeptides, and antifungal use increased, especially in COVID-19 ICUs.

**Conclusions:**

HAI increased during COVID-19. The microorganisms’ susceptibility profile did not change with the pandemic, but the authors observed a disproportionate increase in large-spectrum antimicrobial drug use.

## Highlights

• COVID-19 pandemic was associated with an increase in healthcare-associated infection.
• For the majority of microorganisms, the proportion of resistance did not increase.
• The large spectrum antibiotic consumption increased disproportionately during the pandemic.

## Introduction

The COVID-19 pandemic had a great impact on health services, forcing them to direct efforts and adapt quickly to meet demands. Thus, processes, patient care flows, and demand for supplies such as personal protective equipment, and hand and surface hygiene products, had to be reviewed. In addition, training and guidance for health workers and patients were necessary [1].

The extraordinary demand led hospitals to change routines, potentially generating an increase in infection rates as well as an increase in Multidrug Resistance Microorganisms (MDRO) [2].

Several studies reported an increase in the incidence of MDRO and rates of Healthcare-Associated Infections (HAI), especially device-associated infections [3,4]. However, studies are controversial regarding the impact of the pandemic on HAI rates and microorganisms in non-COVID-19 ICUs [5,6]. Furthermore, few studies analyzed the impact of COVID-19 comparing ICUs dedicated to COVID-19 care and non-COVID-19 ICUs.

Therefore, the goal of this study was to describe the rates of HAI, antimicrobial consumption, and antimicrobial resistance in Intensive Care Units (ICU) in the city of São Paulo, the largest city of Brazil, during the COVID-19 pandemic, and to compare COVID-19 ICUs and non-COVID-19 ICU, as well as compare them with the rates of pre-pandemic period.

## Methods

This retrospective cohort study included all hospitals in the city of São Paulo reporting HAI rates in ICUs from January 2017 through December 2020.

The first COVID-19 case in Brazil occurred on February 25, 2020, therefore the pandemic period was March ‒ December 2020; and the pre-pandemic period was January 2017 ‒ February 2020.

### Setting

São Paulo State Health Department has had a solid HAI surveillance system since 2004 and 96% of hospitals consistently report HAI rates [3]. ICUs report monthly rates of Central-Line Associated Bloodstream Infection (CLABSI), microorganisms isolated from the bloodstream, and antimicrobial susceptibility; mechanical Ventilator-Associated Pneumonias (VAP); and consumption of antimicrobial drugs. In the city of São Paulo since the beginning of the pandemic, in March 2020, hospitals reported data separately for COVID-19-dedicated ICUs and non-COVID-19-ICUs. Definitions of HAI were based on the National Healthcare Safety Network system [7]. The study followed the STROBE checklist for reporting cohort studies.

This is an analysis of secondary data reported by the hospitals to the State of São Paulo Health Authority.

All microorganisms isolated from CLABSI were reported with species identification and antimicrobial susceptibility. Antimicrobial consumption was reported using Daily Defined Doses (DDD)/1,000 patient-days for each drug separately. Data were also grouped by antimicrobial class.

The following rates were evaluated:

CLABSI rate: Number of CLABSI/1,000 Central-Line (CL)-days.
VAP rate: Number of VAP/1,000 Mechanical Ventilator (MV)-days.
CL utilization rate: CL-days/patient-days.
MV utilization rate: MV-days/patient-days.

The proportion of microorganisms causing CLABSI: Number of CLABSI caused by a given microorganism/total number of microorganisms isolated in the period

The proportion of resistance: Number of CLABSI caused by a specific microorganism with a specific resistance profile/total number of the CLABSI caused by the same species. (methicillin-resistant *S. aureus*; vancomycin-resistant *Enterococci;* carbapenem-resistant *A. baumannii, P. aeruginosa*, and Enterobacterales)

Antimicrobial consumption: DDD/1,000 patient-days.

The consistency of the hospitals’ data is systematically checked by the government agency; however, the authors rechecked it. The authors considered data unsuitable if the number of reported microorganisms was lower than the number of CLABSI; if the number of CL-days or MV-days was higher than patients-days; if the hospital did not report continuously all the months in a year after the first yearly notification; or if the variation in DDD was higher than 10-fold over two subsequent months. In cases of data unsuitability, the rate of that hospital for that year was excluded. No hospital had more than one rate per year excluded.

Data regarding hospital funding and the number of beds was obtained from the national register of healthcare services.

Hospitals were stratified by the number of hospital beds (> 150 beds vs. ≤ 150 beds), CL utilization rates during the year 2020 (> 50% vs. ≤ 50%); MV utilization rates during the year 2020 (> 35% vs. ≤ 35%), and type of funding of the hospital (3 categories: private for profit, non-profit private, and public).

### Statistical analysis

Statistical analyses were based on descriptive measurements, time-series plots, and regression models. For rates of CLABSI, and VAP initial analyses were based on time-series plots. In order to have a better understanding of the behavior of trends for CLABSI and VAP, rates were defined and modeled as a function of the number of hospital beds, type of funding, and CL or MV utilization rates. Segmented Poisson regression models were fitted considering the dependent variable the rate (either CLABSI or VAP rates) and as the independent variable time (monthly scale). The segmentation was considered based on a deterministic change point in March 2020 (discriminating between pre-pandemic and pandemic periods). Interpretations for the trends were based on the log scale of rates of infections (additive relationship) as well as on the original scale (multiplicative relationship).

The proportion of microorganisms, the proportion of resistance for specific microorganisms, and DDD were analyzed using Wilcoxon signed-rank test.

The study was approved by the institutional review board (CAE: 38395120.2.0000.0068).

## Results

**Fig. 1:**
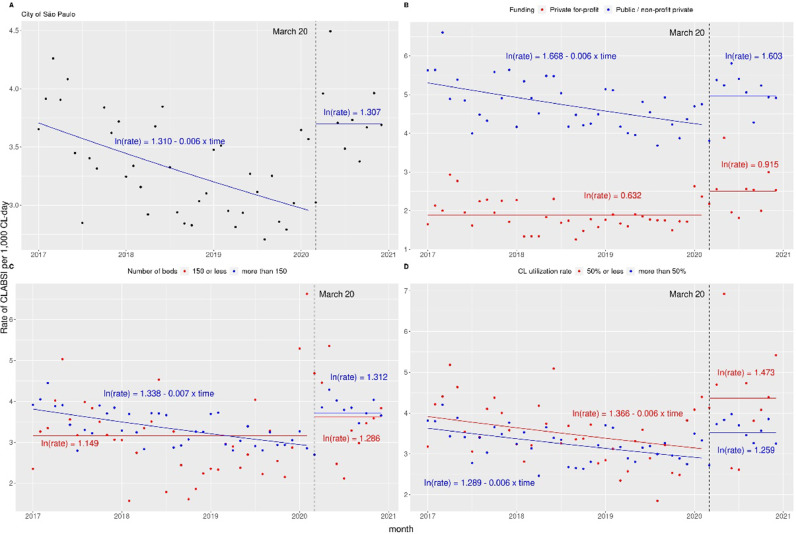
(A) Central-Line-Associated Bloodstream Infections (CLABSI) rates for intensive care units, City of São Paulo, Brazil, 2017‒2021; (B) CLABSI rates according to the type of funding of the hospitals; (C) CLSI rates according to the number of hospital beds; (D) CLABSI rates according to Central Line (CL) utilization rates (Dots represent the observed CLABSI rates and lines represent the adjusted rates).

**Fig. 2:**
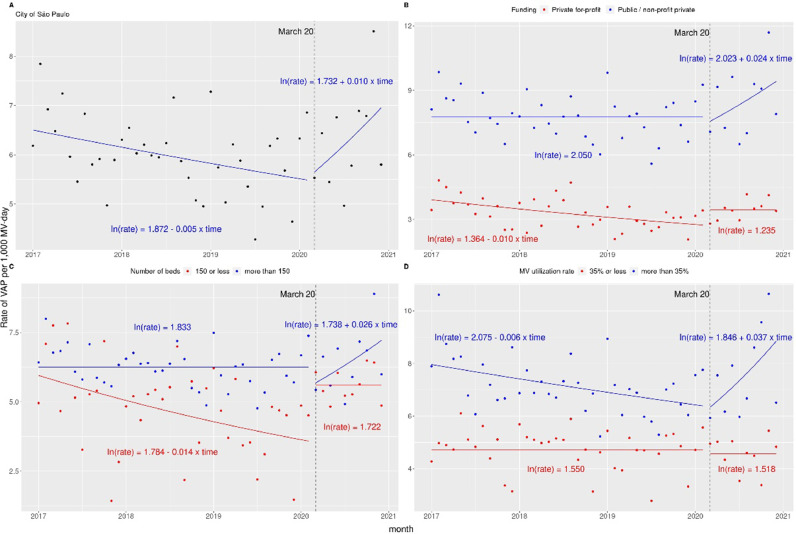
(A) Ventilator-Associated Pneumonia (VAP) rates for intensive care units, City of São Paulo, Brazil, 2017‒2021; (B) VAP rates according to the type of funding of the hospital; (C) VAP rates according to the number of hospital beds; (D) VAP rates according to Mechanical Ventilator (MV) utilization rates (Dots represent the observed CLABSI rates and lines represent the adjusted rates).

The yearly number of hospitals that notified data ranged from 125 to 128. 134 on-COVID-19 ICUs, and 86 COVID-19 ICUs reported infection rates; 62% of hospitals were private for-profit; 58% had > 150 beds, and the median number of ICU beds before the pandemic was 15 (range: 3‒161). The monthly aggregated pre-pandemic CL days ranged from 34,025 to 43,088; and MV days from 15,430 to 24,542 (Supplemental File). HAI rates over time can be seen in Fig. 1, Fig. 2.

### HAI rates in non-COVID-19 ICU

#### Central line-associated bloodstream infections (CLABSI)

CLABSI rates declined during the pre-pandemic period. In January 2017 the rate per 1,000 CL-days was higher for public and non-profit private hospitals, 5.30 (± 0.2) vs. 1.88 (± 0.05) for private for-profit hospitals (p < 0.001). Throughout the pre-pandemic period, the rates declined in public and non-profit private hospitals by 0.61% (± 0.15%) month, while the rates remained stable for private for-profit hospitals (Fig. 1C). In addition, throughout the pre-pandemic period, larger hospitals (> 150 beds) had higher rates compared to smaller hospitals, 3.81 (± 0.11)/1,000 CL-days vs. 3.16 (± 0.11)/1,000 CL-days. Also, CLABSI rates declined estimated at 0.72%/month (± 0.14%) in larger hospitals, while the smaller hospitals presented stable rates (Fig. 1B). Regarding CL-utilization, ICUs with lower CL utilization (≤ 50%) had higher CLABSI rates than institutions with > 50% (p = 0.02) (3.92 [± 0.14]/1000 CL-days vs. 3.63[± 0.10]). Both groups had the same decline in infection rates over the pre-pandemic period (p = 0.24).

After the onset of the pandemic, an increase in CLABSI was observed (Fig. 1A). The increase in rates was statistically significant for larger hospitals (p < 0.001) and marginally significant for small hospitals (p = 0.08). During the pandemic, both categories showed stable rates, estimated at 3.71 (± 0.12)/1,000 CL-days for larger and 3.62 (± 0.25)/1,000 CL-days for smaller hospitals (p = 0.73).

Immediately at the onset of the pandemic, there was a sudden significant increase: 0.62 (± 0.01)/1,000 CL-days (p < 0.001) for private for-profit hospitals, and 0.74 (± 0.07)/1,000 CL-days (p = 0.001) for public/private non-profit hospitals. After that, rates remained stable, with higher rates for public and non-profit private hospitals (4.97 ± 0.18/1,000 CL-days) than for private for-profit hospitals (2.50 ± 0.12/1,000 CL-days).

#### Ventilator-associated pneumonia (VAP)

VAP rates also decreased in the pre-pandemic period (Fig. 2A). The initial estimated rate was 3.91 (± 0.23)/1,000 MV-days which decreased by 0.97% (± 0.29%) per month. Private for-profit hospitals had lower VAP rates during the entire study (Fig. 2B). Larger hospitals maintained a stable rate during the pre-pandemic period whereas smaller hospitals showed a steady decrease (1.36% [±0.38%] month). Hospitals with higher MV use had higher rates initially, that decreased by 0.59% (±0.17%)/month. Public and non-profit private hospitals had higher yet stable pre-pandemic VAP rates.

Starting in March 2020, there was a steady increase in VAP rates. Larger hospitals showed an increase in VAP rates (2.68% [± 1.16%]/month), while in smaller hospitals there was a sudden statistically significant increase to 5.60 (± 0.43)/1,000 MV-days (p < 0.001) after which VAP rates remained stable. Public and non-profit private hospitals VAP rates increased 2.47 % (± 1.22%) per month during the pandemic period. Hospitals with a higher MV use an important and continuous increase in rates during the pandemic (3.77% [± 1.30%] per month).

### Comparison between COVID-19 and non-COVID-19 ICUs

COVID-19 ICUs had higher MV utilization rates (Table 1). CL utilization rates and patient days were similar for both types of ICU.

**Table 1:** Distribution of microorganisms that caused central line-associated bloodstream infections. City of São Paulo, Brazil (January 2017 ‒ December 2020).

	Pre-pandemic period (January 2017 ‒ February 2020)	Pandemic period (March ‒ December 2020)		
		Non-COVID-19 ICU	COVID-19 ICU	p-value comparison between COVID-19 and non-COVID-19 ICU
	n = 5,296 (%)	n = 1,604 (%)	n = 1,509 (%)	
*K. pneumoniae*	1,019 (19%)	293 (18%)	251 (17%)	0.97
*Candida sp*	647 (12%)	202 (13%)	182 (12%)	0.14
*S. aureus*	599 (11%)	184 (11%)	190 (13%)	0.06
*A. baumannii*	523 (10%)	146 (9%)	177 (12%)	0.01
Other Enterobacteriales	474 (9%)^a^	137 (9%)	92 (6%)	>0.99
*Enterococcus sp*	389 (7%)^a^	144 (9%)	220 (15%)	0.0003
*P. aeruginosa*	320 (6%)	109 (7%)	73 (5%)	0.25
*E. coli*	124 (2%)	51 (3%)	21 (1%)	0.39
*Burkholderia/ Stenotrophomonas*	95 (2%)	26 (2%)	20 (1%)	0.33

a < 0.05 when compared to the pandemic period.

The median rate of CLABSI in COVID-19 ICUs was 4.8/1000 CL-days versus 3.3/1000 CL-days in non-COVID-19 ICUs (Supplemental Material).

The rates of VAP did not differ between COVID-19 ICUs and non-COVID-19 ICUs with median rates of 4.1 and 4.5/1,000-MV-days, respectively (Supplemental Material).

### Microorganisms isolated from CLABSIs

During the pre-pandemic period, 5,296 microorganisms were isolated from CLABSI. The most common species was *K. pneumoniae* (18%), followed by *S. aureus* (12%), and *Enterococcus spp*. (12%). During the pandemic 1,509 microorganisms were reported in COVID ICUs, and 1,604 in non-COVID ICUs. Comparing the total proportion in the pandemic period with the pre-pandemic period the authors observed that only *Enterococcus spp*. were more frequent during the pandemic (7% vs. 12%, p = 0.02). Enterobacterales other than *E. coli* and *K. pneumoniae* decreased in the pandemic period (9% vs. 7%, p = 0.02) (Table 2). Comparing COVID-19 ICUS with non-COVID-19 ICUs, the former had a high proportion of CLABSI due to *A. baumannii* and *Enterococcus spp*. (Table 1).

**Table 2:** Distribution of resistance profile of the microorganisms that caused central line-associated bloodstream infections. City of São Paulo, Brazil (January 2017 ‒ December 2020).

Multidrug Resistant Microorganism	Pre-pandemic period (January 2017 ‒ February 2020)	Pandemic period (March ‒ December 2020)		
		Non-COVID-19 ICU	COVID-19 ICU	
	% (n)	% (n)	% (n)	p-value
Carbapenem-resistance in *E. coli*	6% (7/24)	2% (1/51)	14% (3/21)	0.55
Carbapenem-resistance in *K. pneumoniae*	61% (620/1,019)	64% (187/293)	81% (204/251)	0.05
Carbapenem-resistance in *Enterobacter* spp. % (n)	18% (86/474)	23% (32/137)	30% (28/92)	0.62
Carbapenem-resistance in *P. aeruginosa*	44% (140/320)	39% (42/109)	38% (28/73)	0.30
Carbapenem-resistance in *A. baumannii* % (n)	90% (469/523)	88% (128/146)	91% (161/177)	0.04
Vancomycin resistance in *Enterococcus* spp. % (n)	46% (177/389)^a^	31% (45/144)	24% (53/220)	0.21
Methicillin resistance in *S. aureus* % (n)	60% (360/549)^a^	62% (114/184)	58% (110/190)	0.40

a < 0.05 when compared to the pandemic period.

The authors observed an increase in the proportion of vancomycin resistance in *Enterococcus* spp. when comparing the pre-pandemic with a pandemic period (p=0.02) as well as methicillin resistance in *S. aureus*.

During the COVID-19 pandemic, the proportion of carbapenem resistance in *A. baumannii* was higher in COVID-19 ICUs when compared with non-COVID-19 ICUs. The same occurred with carbapenem resistance in *K. pneumoniae* (64% vs. 81%, = 0.05) (Table 2).

### Antimicrobial consumption

The most common antibiotics used in the pre-pandemic period were carbapenems (197.9 DDD/1000 patient-days), followed by ceftriaxone (223.0 DDD/1000 patient-days), and glycopeptides (180.7 DDD/1000 patient-days). Antibiotic consumption in non-COVID ICUs during the pandemic period was similar to the pre-pandemic period except for quinolones that decreased during the pandemic. However, in the COVID-19 ICUs, there was an increase in almost all antimicrobials except for carbapenem and ceftriaxone. The largest increases were for polymyxin, glycopeptides, and echinocandins (Table 3).

**Table 3:** Accumulated DDD/1,000 patient-days in Intensive Care Units of the city of São Paulo, Brazil (January 2017 ‒ December 2020).

Anti-microbial	Pre-pandemic period (January 2017 ‒ February 2020) Accumulate DDD/1,000 patient-days	Pandemic period (March ‒ December 2020)		
		Non-COVID-19 ICU	COVID-19 ICU	p-value
Polymyxins	6.43	6.66	146.20	<0.001
Carbapenem	19.79	19.14	20.50	0.53
Glycopeptide	18.07	20.10	40.14	<0.001
β-lactamase inhibitor + penicillin	14.62	15.04	6.24	<0.001
Ceftriaxone	22.30	22.03	25.74	0.13
Antipseudomonal cephalosporins	2.91	2.25	1.33	<0.001
Quinolones	4.69^a^	3.52	5.20	0.008
Azoles	5.89	4.86	16.25	<0.001
Echinocandins	3.00	4.13	27.63	<0.001

a < 0.05 when compared to the pandemic period.

## Discussion

This longitudinal series of 220 ICUs over a period of five years included the first COVID-19 pandemic year. The authors observed an abrupt increase in CLABSI rates at the onset of the pandemic, then stable yet high rates. VAP rates showed a progressive increase after the onset of the pandemic. The proportion of resistant microorganisms causing CLABSI did not change significantly compared with the pre-pandemic period, especially in non-COVID-19 ICUs, despite the increase in HAI rates. When comparing COVID-19 ICUs with non-COVID-19 ICUs, the only microorganism that increased carbapenem resistance was *A. baumannii.* Antimicrobial consumption increased markedly in COVID-19 ICU mainly due to antifungals, polymyxins, and glycopeptides.

An increase in CLABSI rates during the pandemic was described in other studies [8]. A study from the United States with 148 hospitals, described an increase in CLABSI of 60% over 7 months [2]. Suboptimal nurse-to-patient ratios, barriers to personal protective equipment, lower compliance with hand hygiene, and work overload were potential causes [8]. These hypotheses can also be confirmed by analyzing health systems that were more prepared for the COVID-19 pandemic; the German National Reference Center for Surveillance of Nosocomial Infections did not observe an increase in CLABSI rates during the first year of the pandemic, although a significant increase in the use of central venous catheter was observed; additionally, a study in Singapore reported that measures to prevent nosocomial COVID-19 contributed to reducing overall HAI rates, attributing their success to their previous experience with SARS in 2003 [9,10].

Brazil was particularly affected by the pandemic with a shortage of ICU nurses, intensive care doctors, and physical therapists. A questionnaire involving 1,985 Brazilian healthcare professionals showed burnout in 60%, partially attributed to staffing shortages [11].

A systematic review described that the incidence of VAP in COVID-19 ranged from 21% to 64% [12]. In the present study, VAP rates did not rise immediately after the onset of the pandemic, but progressively. Another Brazilian study also described monthly increases in VAP during the pandemic [13]. Several features may explain this increase, first, patients with severe COVID-19 usually have long ICU stays on mechanical ventilation. Pronation, frequently used in COVID-19, may impair adherence to preventive measures, such as bed elevation and oral hygiene [14]. Furthermore, the intensive use of corticosteroids and other immunomodulatory drugs could increase the risk of VAP, as demonstrated in a large French cohort that included more than 3,000 patients with COVID-19 admitted to the ICU [15].

Sub-analyses were done to understand the impact of the pandemic on HAI in different settings. The increase in rates was more important in non-profit/public and large hospitals. Before the pandemic, CLABSI and VAP rates were continuously decreasing under a state-wide prevention government program [16]. In Brazil, the national health system (SUS) is responsible for 75% of healthcare. Hospitals that serve SUS (public and non-profit private) received the burden of the crisis. Additionally, SUS hospitals usually have lower healthcare professional-patient ratios and worse structural conditions [17]. The larger hospitals had higher rates of CLABSI and VAP.

Rates of VAP behaved differently according to MV utilization. Hospitals with higher utilization rates presented significant and continuous increases in VAP rates. The authors first hypothesized that this difference was due to the fact these units were responsible for assisting COVID-19 patients. However, the comparison between COVID-19 and non-COVID-19 units showed similar rates. Thus, the authors believe the higher increase in VAP should be attributed to work overload and to mechanical ventilation itself.

On the other hand, CLABSI rates increased faster in units with lower CL. Low CL utilization may reflect less severe patients, and the authors believe that during the health crisis health professionals with the lowest experience were directed to these ICUs.

The majority of healthcare surveillance studies did not report rates separately for COVID-19 ICUs, however, in the city of Sao Paulo the authors could compare units [18,19]. Surprisingly VAP rates were similar between COVID-19 and non-COVID-19 ICUs, but CLABSI rates were higher in COVID-19 ICUs.

Infection rates increased during the pandemic, however, the distribution of microorganisms changed only slightly with an increase of CLABSI due to *Enterococcus*. Compared to non-COVID-19 ICUs, COVID-19 ICUs had more infections due to *A. baumannii* and *Enterococcus* spp. Additionally in the COVID-19 ICU carbapenem resistance was higher among *K. pneumoniae* and *A. baumannii*.

An increase in MDRO during pandemic was described in several studies. A multicentric Italian study reported that 46% of COVID-19 patients developed HAI, and 35% of them were caused by an MDRO [20]. Data from two different Brazilian states showed a >108% increaseis in carbapenem-resistant *Acinetobacter* [13,21]. In this study the authors did not observe an increase in *A. baumannii* when compared with the pre-pandemic period. What really increased was the absolute number of MDRO infections. Furthermore, the use of drugs against MDRO such as polymyxins and vancomycin increased greatly.

The authors believe that the increase in absolute numbers of infections led to the perception that MDRO as a problem had increased and was associated with the high mortality of COVID-19 patients. This led to a disproportionate increase in drugs used to treat MDRO. Several studies described an increase in antimicrobial consumption in COVID-19 patients. One study found that 57% received antibiotics on hospital admission, although only 3.5% had confirmed bacterial infections [22]. A Scottish point prevalence survey found that 62% of COVID-19 patients received antimicrobial drugs on hospital admission, and 46% of critical patients were using antibiotics during the survey [23]. A meta-analysis that included 154 studies reported that 62% of COVID-19 patients used antibiotics [24].

Physicians have good reason to prescribe empiric antimicrobials: 24% of hospitalized COVID-19 patients, and 45% of those on mechanical ventilation will develop a secondary infection [25]. Furthermore, mortality is 21% higher in patients receiving inadequate empirical antimicrobial therapy [26]. Therefore, the balance between antimicrobial overuse and delay in starting effective treatment is difficult and calls for active antimicrobial stewardship programs.

The main strategy for controlling antimicrobial consumption in critical situations such as the COVID-19 pandemic is the understanding of local HAI rates and microbiology. Identifying factors associated with infection, susceptibility, and the use of antimicrobials during the pandemic also helps to target the problem, especially in scenarios of limited resources.

The main limitation of this study is its ecological design. Furthermore, the use of secondary data increases the risk of inconsistencies. To reduce this, the authors made a critical per-hospital data analysis and excluded data with inconsistencies. Conversely, a strong point was the evaluation starting several years before 2020, which allowed us to define the trend of the HAI rates before COVID-19.

In conclusion, the COVID-19 pandemic led to an increase in HAI. Hospitals with lower resources as well as reference services are more vulnerable to this kind of catastrophe. The present study demonstrated an outstanding increase in broad-spectrum antimicrobial consumption that was disproportionate to the increase in MDRO infections. Infection control professionals and health care agencies should be aware of this possibility and act toward the prevention of infections and their treatment.

## Authors’ contributions

MPF: Collected and interpreted data, wrote the first draft of the manuscript.

DBA: Collected and interpreted data, wrote the first draft of the manuscript.

BMT: Collected and interpreted data.

VOCB: Collected data.

IM: Collected data.

MSL: Collected data.

ARG: Wrote the first draft of the manuscript.

GM: Collected and interpreted data.

MSO: Collected and interpreted data, wrote the first draft of the manuscript.

ACPL: Did the statistical analysis.

ASL: Interpreted data and did the critical revision of the manuscript.

## Declaration of Competing Interest

The authors declare no conflicts of interest.

## Appendix Supplementary materials

Image, application 1
