# Supplementary material for: Impact of COVID-19 on healthcare-associated infections: Antimicrobial consumption does not follow antimicrobial resistance
Source: Clinics (Sao Paulo). 2023 Jun 13;78:100231. doi: 10.1016/j.clinsp.2023.100231 (PMC10261712; doi:10.1016/j.clinsp.2023.100231)
Supplement: Supplementary file 1 [file mmc1.docx]

**CLINICS-D-22-00659_ Supplemental File and Material**

**Supplemental File and Material**

**Supplement Table** Characteristics of the hospitals of the city of São Paulo reporting healthcare-associated infections in Intensive Care Units (ICU) to the São Paulo State Health Department (2017‒2020).

| **Hospital characteristics** | **COVID-19 ICU** | **Non-COVID-19 ICU** |
| --- | --- | --- |
|  | **n=86 (%)** | **n=134 (%)** |
| Funding |  |  |
| Public | 33 (38%) | 44 (33%) |
| Non-profit private | 6 (7%) | 11 (8%) |
| Private for-profit | 47 (55%) | 79 (59%) |
| Number of hospital beds ‒ mean (SD) | 239 (126) | 208 (111) |
| Number of ICU beds range | 8‒200 | 3‒161 |
| Mechanical ventilator utilization rate in year 2020 ‒ mean (SD) | 46% (15) | 31% (14) |
| Mechanical ventilator-days during year 2020 ‒ mean (SD) | 2,143 (1,557) | 1,688 (1,227) |
| Central line utilization rate in year 2020 – mean (SD) | 61% (15) | 54% (18) |
| Central line-days during year 2020 – mean (SD) | 2,918 (2,113) | 3,138 (2,406) |
| Patient-days during year 2020 – mean (SD) | 4,844 (3,327) | 5,376 (3,646) |

SD, Standard Deviation.

**Supplement File** Monthly central line-associated bloodstream infection (CLABSI) rates (A) and Ventilator-Associated Pneumonia (VAP) rates (B) in Intensive Care Units (ICU) in the city of São Paulo (2017‒2020). During the pandemic period (starting in March 2020) rates are presented separately for COVID and non-COVID ICUs.

**
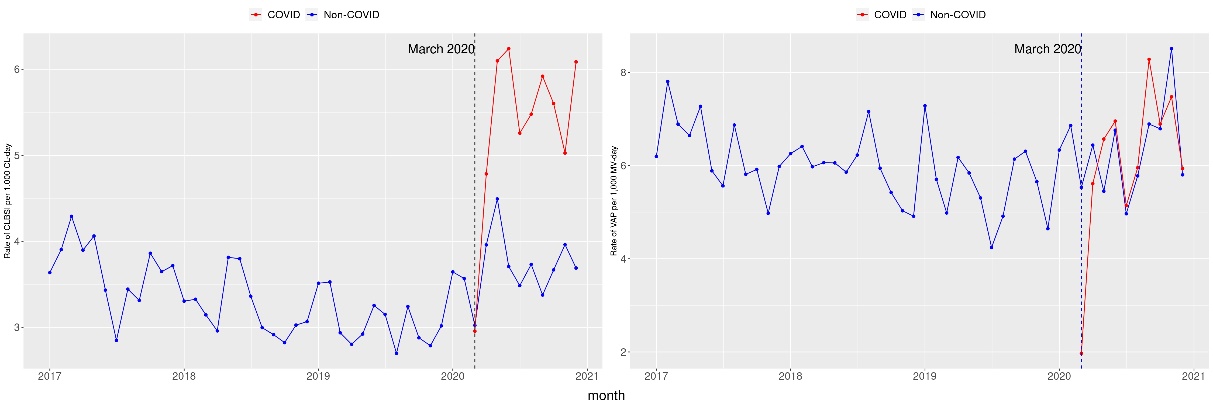
**
